# The SPHERE Study. Secondary prevention of heart disease in general practice: protocol of a randomised controlled trial of tailored practice and patient care plans with parallel qualitative, economic and policy analyses. [ISRCTN24081411]

**DOI:** 10.1186/1468-6708-6-11

**Published:** 2005-07-29

**Authors:** Andrew W Murphy, Margaret E Cupples, Susan M Smith, Molly Byrne, Claire Leathem, Mary C Byrne

**Affiliations:** 1 Department of General Practice, Clinical Sciences Institute, National University of Ireland, Galway, Ireland; 2 Department of General Practice, Queen's University, Dunluce Health Centre, 1 Dunluce Avenue, Belfast BT9 7HR, Northern Ireland; 3 Department of Public Health and Primary Care, Trinity College Centre for Health Sciences, Adelaide and Meath Hospital, Tallaght, Dublin 24, Ireland; 4 Department of Psychology, National University of Ireland, Galway, Ireland

## Abstract

**Background:**

The aim of the SPHERE study is to design, implement and evaluate tailored practice and personal care plans to improve the process of care and objective clinical outcomes for patients with established coronary heart disease (CHD) in general practice across two different health systems on the island of Ireland.

CHD is a common cause of death and a significant cause of morbidity in Ireland. Secondary prevention has been recommended as a key strategy for reducing levels of CHD mortality and general practice has been highlighted as an ideal setting for secondary prevention initiatives. Current indications suggest that there is considerable room for improvement in the provision of secondary prevention for patients with established heart disease on the island of Ireland. The review literature recommends structured programmes with continued support and follow-up of patients; the provision of training, tailored to practice needs of access to evidence of effectiveness of secondary prevention; structured recall programmes that also take account of individual practice needs; and patient-centred consultations accompanied by attention to disease management guidelines.

**Methods:**

SPHERE is a cluster randomised controlled trial, with practice-level randomisation to intervention and control groups, recruiting 960 patients from 48 practices in three study centres (Belfast, Dublin and Galway). Primary outcomes are blood pressure, total cholesterol, physical and mental health status (SF-12) and hospital re-admissions.

The intervention takes place over two years and data is collected at baseline, one-year and two-year follow-up. Data is obtained from medical charts, consultations with practitioners, and patient postal questionnaires.

The SPHERE intervention involves the implementation of a structured systematic programme of care for patients with CHD attending general practice. It is a multi-faceted intervention that has been developed to respond to barriers and solutions to optimal secondary prevention identified in preliminary qualitative research with practitioners and patients. General practitioners and practice nurses attend training sessions in facilitating behaviour change and medication prescribing guidelines for secondary prevention of CHD. Patients are invited to attend regular four-monthly consultations over two years, during which targets and goals for secondary prevention are set and reviewed. The analysis will be strengthened by economic, policy and qualitative components.

## Background

Coronary heart disease (CHD) is a common cause of death and an important cause of morbidity in Ireland. Secondary prevention of heart disease involves long-term management of risk factors among people who have been diagnosed with established CHD. The Scottish Intercollegiate Guidelines Network has defined secondary prevention as the 'identification and modification of risk factors by the introduction of lifestyle measures and pharmacological therapy and cardiac rehabilitation' [1]. Secondary prevention has been recommended as a key strategy for reducing levels of CHD [2,3]. Secondary prevention can be achieved by stopping smoking, making healthier food choices (including reducing fat intake and increasing intake of fruit, vegetables and fibre), becoming physically active, achieving an ideal weight, consuming alcohol in moderation, appropriate prescription of, and adherence to, pharmacological therapy, achieving blood pressure level at or under 140/90 mmHg and achieving a total cholesterol level at or below 5 mmol/l [3].

Most people with CHD regularly attend their general practitioner and general practice has been highlighted as an ideal setting for secondary prevention initiatives [2]. Previous studies of provision of secondary prevention in general practice have identified sub-optimal levels [4-6]. Current indications suggest that there is considerable room for improvement in the provision of secondary prevention for patients with established heart disease on the island of Ireland [7]. Recent data from the national Heartwatch programme suggest that 44% of Irish patients with established heart disease have a systolic blood pressure above the recommended guidelines of 140 mmHg, and that 36% have a baseline cholesterol level of greater than 5 mmol/l (Leahy J, personal communication). In a survey of secondary preventive care of 1,600 patients, from 35 randomly selected general practices in the west of Ireland, results were as follows: 23% of patients were regular smokers, 45% had cholesterol readings greater than 5 mmol/l and 34% had blood pressure readings greater than 140/90 mmHg. In addition, GP records were found to be incomplete, with 38% of patients with no record of smoking status and 25% with no cholesterol reading. Information from Northern Ireland suggests a similar situation: a recent survey found that among patients with a confirmed diagnosis of CHD 18% smoked, 25% had a body mass index (BMI) greater than 30, 51% had a systolic blood pressure greater than 140 mmHg and approximately 50% had cholesterol levels greater than 5 mmol/l [8].

Randomised controlled trials have investigated the effectiveness of secondary prevention interventions including health visitor advice [9], nurse-led secondary prevention clinics [10,11], outreach visits to practices by nurses trained in cardiovascular risk factor recording [12], provision of specialist liaison nurses to bridge the gap between secondary and primary care [13], postal prompts to patients to encourage lifestyle changes [14], audit and systematic recall of patients from disease registers [15], combined training to all practice staff in information systems and evidence-based medicine [16] and a cognitive-behavioural disease management programme [17].

These trials have reported a number of successful outcomes, including increased physical activity [9,10,17], improved diet [9,10,17], increased health functioning [9,10,17], increased patient assessment and recording of risk factors [12,14-16], increased rate of consultation for coronary heart disease [14], improvements in medication prescribing [10,15], improved lipid management [10,14,16], reduction in cholesterol level [16], improved blood pressure management [10], increased lifestyle advice provision [14] and reduced anxiety and depression [17].

However, with the exception of the PIER trial [16] which achieved significant reductions in cholesterol levels among intervention group patients, trials have not achieved significant improvements in objective biophysical risk factors, such as blood pressure or cholesterol levels.

A systematic review identified twelve randomised trials of disease management programmes for patients with established heart disease [18]. It concluded that such programmes *do *improve processes of care, reduce hospital admissions and enhance quality of life and functional status in patients with CHD. However, the reviewers noted that studies included imprecise descriptions of both the interventions and the usual care provided to the control groups, resulting in an inability to determine the incremental benefits of the various components of each intervention. Reviewers concluded that several important issues require further clarification, including the optimal mix of interventions and the cost-effectiveness or the economic impact of such interventions. Another earlier systematic review [19] also noted both the lack of detail provided of complex health service interventions, especially in the description of the usual care provided to control groups, and the need for the careful design and evaluation of different implementation models of secondary prevention.

Further possible explanations for the lower than expected clinical impact of secondary prevention interventions to date include inadequate consideration of doctors' and patients' perspectives on heart disease, lack of patient-centredness of the intervention and a failure to tailor practice interventions to individual patients and practices [13]. Wiles [20], utilising a qualitative approach as part of the SHIP study, especially emphasised the failure of 'official accounts' to acknowledge patient perceptions as a key feature in people's reluctance to adopt lifestyle change.

There is substantial evidence that structured systematic care is important to improve levels of secondary prevention of coronary heart disease, and an effective register, recall system and routine audit of care are essential components of such a system [13,21]. There is also some evidence to suggest that nurse-led clinics [10], cognitive behavioural interventions [17,22,23] and tailored training identified by practice staff [16] are effective in improving secondary prevention in general practice and facilitating patients to make lifestyle changes.

A recent Cochrane review [24] of interventions to implement prevention in primary care highlighted the need to tailor interventions to address specific barriers to change in a particular setting. The review also concluded that multi-faceted interventions may be more effective than single interventions, as more barriers to change can be addressed. The authors of the review recommend that future research should analyse barriers to change and report interventions to implement preventive services in more detail, to clarify how interventions relate to specific barriers. Since such complex interventions are likely to be more costly than single interventions, these reviewers also advise that economic evaluations should be included in future studies.

The SPHERE Study aims to take these recommendations on board in developing an intervention to improve secondary prevention of CHD in general practice in Ireland. The SPHERE study involves the implementation of a structured systematic programme of care for patients with CHD attending general practice. The SPHERE intervention is multi-faceted and has been developed to respond to the barriers and solutions to optimal secondary prevention identified in preliminary qualitative research with practitioners and patients. The delivery of the intervention is tailored as much as possible to the needs of practices and patients. The process of the intervention will be documented and described in sufficient detail to allow replication of the intervention and to enable identification of effective intervention components. The intervention will be implemented over a 24 month period, with data collection at baseline, 12 months and 24 months. Data collection at these time points will enable identification of any initial and/or sustained changes effected by the intervention. An economic evaluation of the intervention will be carried out. The implementation of the intervention will be monitored throughout the duration of the trial and documented.

In summary, SPHERE will add to what is already known in respect of secondary prevention in primary care, i.e.

• that structured programmes, with continued support and follow-up of patients, help improve recording of process measures of preventive care, patients' functional status, quality of life and lifestyle change [9-11,15,17,25].

• prompts to patients improve their attendance but there is no associated change in measures of risk [13,15].

• structured recall programmes which do not take account of individual practice needs improve recording but not prescribing [15]; provision of training, tailored to practice needs of access to evidence of effectiveness of secondary prevention is associated with improved prescribing [16]

• patient centred consultations impact positively on health outcomes [26] but must be accompanied by attention to disease management guidelines to achieve clinical objectives [27].

SPHERE will:

• record organisational detail of care, in both intervention and control practices

• provide information and training tailored to practices' needs for delivery of secondary prevention

• support patient centred consultations and practitioners' adherence to disease management guidelines.

## Aim

To design, implement and test tailored practice and personal care plans to improve the process of care and objective clinical outcomes for patients with established coronary heart disease in general practice in Ireland.

## Design

Cluster randomised controlled trial, with practice-level randomisation to intervention and control groups.

## Random Selection of Practices and Patients

### Practice identification (48 practices, 16 at each centre)

The same method is followed by each centre in Belfast, Dublin and Galway for their respective regions of the Eastern Health and Social Services Board, the South Western Area Health Board and the Western and North Western Health Boards.

Lists of all practices meeting eligibility criteria are generated by the research nurses in each centre (see 'practice eligibility' section below for criteria). Practices are then allocated a number and randomly selected from the lists in each region by an individual independent of the research team using computer-generated random numbers. The practice manager or lead general practitioner in each practice is contacted by telephone by the research nurse to determine their interest in receiving information about the trial. With their agreement, information is posted to the practice (see additional file 1: Appendix A) and arrangements are made for a member of the research team to visit the practice to discuss their participation in the study.

During this meeting the research nurse explains the study to practice staff and gets commitment to the study, indicated by a signature, from practice staff (at least one GP, one practice nurse and one administrator). A Practice Implementation Plan is completed (see additional file 1: Appendix A) and all practice staff are invited to review and approve it. Practices that agree to participate are offered an honorarium of €1000 or the Sterling equivalent upon completion of the study.

If selected practices decline to participate or confirm that they do not meet the eligibility criteria, the next practice on the list is selected. Relevant details (number of whole-time equivalent (WTE) practitioners, list size and reason for not being recruited or not agreeing to participate) are recorded for those practices which are not recruited.

### Patient identification (20 patients in each practice)

Participating practices compile a list of all eligible patients (see 'patient eligibility' section below for detail) from their practice and allocate a number to each patient from 1 to Y (where Y is the total number of patients on the list). Research nurses assist the practices in using a list of random numbers to select patients for participation (see additional file 1: Appendix A). A confidentiality agreement will be signed between research nurses and practice staff, to ensure that normal ethical procedures governing each practice are adhered to. Selected patients are posted an invitation letter, information sheet and reply slip (see additional file 2: Appendix B) as well as a questionnaire (see additional file 3: Appendix C). Patients who do not respond within ten days are contacted by a member of the practice staff to ascertain interest in participation. If selected patients do not confirm that they fulfil the eligibility criteria or decline to participate, the next patient on the list is selected until the quota of 20 is reached. The age, gender and diagnostic inclusion criteria for those who do not participate are recorded. Patients who agree to participate are invited to attend the practice for a baseline data collection consultation.

Once sixteen practices have been recruited and the relevant patient baseline data collected, at each centre an individual independent of the research team using computer-generated random numbers will allocate, with prior stratification, practices to intervention or control groups. Stratification will be according to the WTE for practitioners (less-than-two and two-or-more). Patient identification and baseline data collection occur prior to practice randomisation to minimize potential bias introduced by researchers and practitioners being aware of their allocation to either the intervention or control group. For intervention practices, meetings will be arranged to develop tailored practice care plans, deliver appropriate training, and support practitioners in reviewing patients four monthly for two years. Control practices will be visited again after one year and after two years, at which time points follow-up data will be collected.

## Practice and Patient Selection: Inclusion and Exclusion Criteria

### Practice eligibility

Practices are eligible to take part if they:

• Have a practice nurse, involved in general patient care (to be confirmed at the initial practice visit).

• Did not participate in the pilot phase of the study.

#### For practices in the Republic of Ireland

• Are not participating in Heartwatch (a national pilot programme in the Republic of Ireland on the provision of secondary care in general practice, currently ongoing).

• Have a minimum General Medical Scheme (GMS) list size of 700 patients. Based on an Irish prevalence rate of 3.16% for CVD [7], this will ensure at least 22 eligible GMS patients per practice, a figure which will be supplemented by non-GMS patients.

#### For practices in the North of Ireland

• Have a minimum NHS list size of 1800 patients. Based on a UK prevalence rate of 2.6% for CVD [4], this will ensure at least 40 eligible patients per practice.

### Patient eligibility

#### Inclusion criteria

Patients with existing cardiovascular disease (defined as: documented MI, CABG or angioplasty, or a diagnosis of angina – confirmed by exercise stress test, isotope test or coronary angiogram) are eligible for inclusion.

#### Exclusion criteria

Patients with significant mental or physical illness (based on the recorded judgement of practice staff), which is likely to impair capacity to change lifestyle behaviour or assimilate new information, are not included.

## Sample Size Calculation

**Figure 1:**
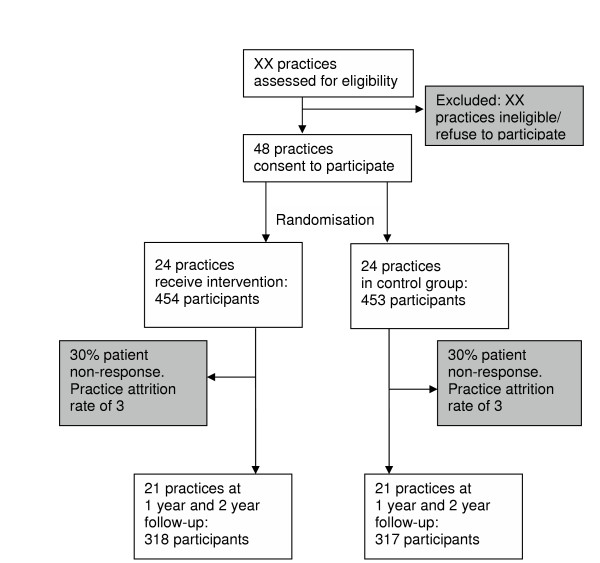
Flow of Practices and Patients Throughout Trial.

The study aims to achieve a final sample of 635 patients from at least 42 practices (see Figure 1).

Sample size calculations are based on the following:

• Power of 80% and alpha of 5%.

• Only patients agreeing to participate in the study and completing baseline data collection will be entered into the study. There will, therefore, be 100% uptake at the start of the study. Reasons for non-participation will be documented and recorded, and participation rates will provide a measure of the acceptability of this type of intervention to patients.

• A final completion rate of 70% is anticipated based on similar studies in the West of Ireland 7 (completion rate = 68.7%) and Dublin [28] (completion rate = 93%).

• Practice attrition of 12.5% has been allowed for (though practice attrition in another similar study was only 4% [28]).

• Improvements in the control group as a result of participation in a research study have been anticipated [25].

• Design effects are calculated using intracluster correlation coefficients from data from similar populations in Galway and Scotland (Campbell N, personal communication).

A total sample size of 907 patients recruited from 46 practices gives a varying but minimum power of 80% to detect significant changes in proportions with poor blood pressure and cholesterol control, physical and mental well being (SF-12) and hospital readmissions at two years. (Detailed power calculations are given below). This allows for a final patient follow-up and retention rate of 70% and a practice attrition rate of 10%. The number of practices is rounded up from 46 to 48, to allow equal numbers of practices to be recruited in each of the three study centres. Sixteen practices will therefore be recruited in each of the three centres, eight intervention practices and eight control practices. Twenty patients are recruited in each practice. Final data analysis will incorporate modelling techniques that are more statistically efficient and will ensure greater power to detect significant changes in study outcomes.

## Power Calculations – Further Detail

### Blood pressure

44% of Irish patients with established heart disease have a systolic blood pressure above recommended guidelines (SBP >140 mmHg) (Leahy J, personal communication). Previous research indicates that an improvement of 20% can be anticipated in the control group [10]. Taking this into consideration, a sample size of 408 gives 80% power to detect a 50% reduction in the proportion of patients with a SBP above 140 mmHg (i.e. giving a proportion of 22%) in the intervention group with a corresponding 20% improvement in the control group. Other studies have indicated that it is possible to achieve similar targets in general practice populations with established coronary heart disease [15]. To take account of cluster randomisation, based on recruiting 15 patients per cluster, a design effect size of 1.15 and an intracluster correlation coefficient of 0.011 [4,10] suggests a sample size of 470 patients from 31 practices. Allowing for a final response rate of 70% and a practice attrition rate of 10% requires recruitment of 670 patients from 34 practices.

### Cholesterol

36% of Irish patients have a baseline cholesterol level above the upper recommended limit, i.e. greater than 5 mmol/l (Leahy J, personal communication). To demonstrate an improvement of 50% in the intervention group and 20% in the control group requires a sample size of 500 patients. To ensure a final sample of 15 patients per practice, this is inflated by a design effect size of 1.27 (ICC = 0.019; Campbell N, personal communication) to give a sample of 635 patients from 42 practices. This is increased to 907 patients from 46 practices to allow for final follow up and response rate of 70% and practice attrition of 10%.

### SF-12

A 5 point improvement in the SF-36 has been found to be clinically significant [29]. Based on a mean at baseline of 53.98 (SD 8.39) [7], a sample size of 120 patients is sufficient to detect a clinically significant improvement of 5 points in the SF-12 measure of physical and mental health status in the intervention group. The intracluster correlation coefficient from previous data [7] is <0.0001, indicating that there is no clustering effect for this variable and that the design effect size is 0. Allowing for a patient retention rate of 70% and a 10% practice attrition rate, 170 patients from 10 practices are needed.

### Hospital re-admissions

Northern data (Cupples M, personal communication) indicates that 43% of control patients had hospital admissions in two years. To reduce this by 50% in the intervention group and 20% in the control group needs a sample size of 356 patients. To ensure a final sample of 15 patients per practice, this is inflated by a design effect size of 1.08 (ICC = 0.006) to give a sample of 406 patients from 27 practices, increased to 580 patients from 30 practices to allow for final follow up and response rate of 70% and practice attrition of 10%.

The study acknowledges that smoking is a significant risk factor for cardiovascular disease. For pragmatic reasons smoking has not been used to power the study, as levels of smoking have become low among this population and its measurement would require a sample size too large for the resources available.

## Intervention

### Level 1: Tailored practice care plans

#### (1) Training for practice staff

Training will be delivered independently in each of the three regional study centres. All trainers will adhere to a single training protocol to ensure standardised delivery of the training across centres. Training delivery will be planned and rehearsed jointly by all trainers using role-play and peer review techniques. In addition, the project manager will act as an observer during the first two training sessions in each centre and will provide feedback to trainers with a view to further standardizing the training. The Irish College of General Practitioners (in the Republic of Ireland) and the Northern Ireland Medical and Dental Training Agency (in Northern Ireland) have approved the proposed training sessions for their educational contribution to practitioners' personal development plans.

##### First training session: Medication training

GPs and practice nurses are invited to attend a ninety-minute training session on medication guidelines, delivered by the study GP in each region:

• Belfast: Dr. Margaret Cupples, Department of General Practice, Queen's University Belfast.

• Dublin: Dr. Susan Smith, Department of Public Health and Primary Care, Trinity College Dublin.

• Galway: Professor Andrew Murphy, Department of General Practice, NUI Galway.

This is an interactive session in which practice staff are invited to review and discuss the most recent medication prescribing guidelines for secondary prevention. The session uses case-based scenarios to enable the practitioners to reflect on their own practice. Practitioners are offered a summary sheet of prescribing guidelines and a summary 'prompt' showing commonly used drugs and dosages within the various medication categories. If practices wish their practice nurse to make prescribing decisions the research nurse will help to prepare appropriate specific protocols.

The objectives of this session are:

• To increase confidence and competence regarding prescribing and the secondary prevention of heart disease.

• To discuss the role of the SIGN guidelines and apply to real clinical situations.

• To explore practitioner attitudes to secondary prevention and to discuss points raised specifically by each practice.

• To address issues of patient adherence to medication.

##### Second training session: Behaviour change training

GPs and practice nurses are invited to attend a ninety-minute training session on facilitating patients with behaviour change, delivered by the research nurse in each region. This is an interactive session in which practice staff are invited to reflect on their views around lifestyle change and practice new techniques (through role play) to improve their ability to facilitate patients with behaviour change. Strategies to increase patient motivation are discussed, following guidelines from brief motivational interviewing literature [30,31]. Additional techniques, based on principles of behaviour modification and social learning theory [32], include: setting small achievable goals, action planning, using prompts, self-monitoring, offering rewards, habit reinforcement, and relapse prevention.

The objectives of this session are:

• To enable practitioners to develop skills based on leading behaviour change theories, which may help them with behaviour change consultations.

• To introduce the SPHERE patient-held booklet and the patient care plan (see 'Level 2' below).

#### (2) Responding to individual practice needs

Additional practice needs in relation to the delivery of preventive care for patients with established cardiovascular disease, as well as the day-to day running of the study, are recorded on a tailored practice care plan and followed up by the SPHERE research nurse.

#### (3) Ongoing support from the SPHERE research nurse

The SPHERE research nurse maintains regular contact with the practices, and is easily contactable by phone if practice staff have any queries about SPHERE.

#### (4) SPHERE newsletter

Intervention practices receive a study newsletter which is published every four months. The newsletter contains the latest news and updates relating to the study.

### Level 2: Tailored patient care plans

#### (1) Initial target setting consultations

The first patient intervention consultation with the GP/practice nurse takes place as soon as possible after baseline data collection, once training of practice staff has been completed. BP and BMI are measured, diet, smoking and exercise habits reviewed and the result of the baseline cholesterol assay discussed. The patient and practitioner together identify areas of management which could be improved and the patient is invited to prioritise one particular aspect of their lifestyle for change. Possible ways of achieving targets reflecting optimal management (BP <140/90 mmHg, cholesterol <5 mmol/l, BMI <25, non-smoking, taking exercise for 30 minutes 5 days per week, eating fruit/vegetables daily and avoiding saturated fat intake, avoidance of stress) are identified. Plans for action are recorded on a personal care plan tailored for each patient and retained in the practice. The GP may also see the patient at this visit or at a further appointment within 2 weeks to determine if any change in medication is appropriate. A further follow up telephone call is made by the practice nurse to give support and address any questions 2 weeks later.

#### (2) The patient-held booklet

This booklet contains information on all the key risk factors for CHD. It is used by the practitioners in all initial target setting discussions with the patient and is given to the patient to be kept by them. The booklet can be referred to at subsequent consultations with the GP or practice nurse for consolidation of information relevant to secondary prevention and cardiovascular risk. There are 6 sections in the book:

a) Medications

b) Smoking

c) Exercise

d) Healthy eating

e) Stress

f) Community support

By providing a summary of lifestyle advice for secondary prevention of CHD, the booklet serves not only as an information resource for patients but also as a reference guide to help practitioners in their lifestyle-related consultations with patients.

#### (3) Regular consultations with patients for two years

Patients are invited to attend for an appointment with the GP/nurse every 4 months. At each visit a review of factors relating to targets and goals for optimal secondary prevention are made as appropriate. Goals relating to diet, smoking and exercise habits are routinely reviewed. Cholesterol is checked if it has been found to be raised at baseline, but if normal at baseline cholesterol is checked annually. Relevant measurements taken at each review visit are recorded and held in the practice and reviewed with the patient at subsequent review visits. Care which would be given according to current clinical guidelines will also be provided, as required.

## Control Practices

Data are collected for patients in intervention and control practices in identical ways. In control practices, data are collected at baseline, one and two years post baseline. Otherwise, patients in control practices continue to receive health care as usual and the nature of this in each participating practice will be clearly described.

## Informed Consent

An information sheet is sent to all potentially eligible patients (see additional file 2: Appendix B). Patients are asked to sign and return, with their postal questionnaire, a reply slip (see additional file 2: Appendix B) to allow further contact regarding the research. At their first consultation for baseline data collection, the GP/practice nurse ensures their understanding of the study, allows them the opportunity to ask questions and confirms their willingness to participate in the study before asking them to sign a consent form (see additional file 2: Appendix B).

## Ethical Approval

Ethical approval has been granted by the Ethics Committee of the Irish College of General Practitioners and the Queen's University Research Ethics Committee.

## Data Collection

Data collection will involve three components:

• **Questionnaire **posted to patient from the practice with administrative assistance from the SPHERE research nurse. Questionnaire is posted back to the practice in a pre-paid envelope. The research nurses liaise with practice staff to follow up non-responders.

• **Consultation **data gathered by practice staff and inserted into one page SPHERE study baseline measurement record.

• **Chart search **to establish process of care and collect patient outcomes recorded in GP records. This is carried out by research nurse (after obtaining patient consent), and recorded onto SPHERE study database.

The above data will be collected at three time points:

1) Baseline

2) One year

3) Two years.

Data are collected at one year to assess the initial effects of the intervention. No results of any interim analyses will be revealed to any member of the research team unless the Data Monitoring Committee (see below) determine that it is unethical to continue the study.

Data to be collected are described below.

**Questionnaire components **(see additional file 3: Appendix C)

A. Physical and mental well-being: SF-12.

B. Exercise: Godin Leisure-Time Exercise Questionnaire.

C. Smoking status: adapted from SLÁN study [33].

D. Diet: DINE, including question regarding portions of fresh fruit and vegetables.

E. Personal and demographic variables:

a. Demographics: name, date of birth, marital status, educational level, occupation, GMS eligibility (for practices in the Republic of Ireland).

b. Economic analysis questions: journey time to GP and hospital.

c. Health service attendance in previous 12 months: including hospital outpatient services, admission to hospital as an inpatient, visits to Accident and Emergency department and days spent as inpatient.

F. Adherence to medication: Medication Adherence Report Scale MARS-5.

**Consultation/physical assessment components (taken by practice nurse/GP) **(see additional file 4: Appendix D)

1. Blood pressure.

2. Total serum cholesterol concentration (HDL and LDL).

3. Current medications, dose and contraindications.

4. Height in cm/weight in kg/BMI/waist circumference in cm/waist hip ratio.

**Chart search data (collected by SPHERE research nurse) **(see additional file 4: Appendix D)

1. Cardiac history: months since diagnosed with CHD, record of myocardial infarction and time since myocardial infarction, presence of angina, investigation confirming diagnosis, previous PTCA or CABG, presence of diabetes.

2. Number of GP and practice nurse consultations in last 12 months.

3. Number of attendances at outpatients/inpatient services in last 12 months.

4. Recording of risk factors in medical records: smoking, cholesterol and blood pressure.

## Data Analysis

### Description of study participants

Practices and patients participating in the SPHERE study will be heterogeneous. Participating practices will be described in terms of:

• Number and gender of full time equivalent GPs

• Number of practice nurses

• Numbers of support staff

• Practice list size – in the Republic of Ireland this will be presented as GMS list size and estimated non-GMS list.

Participating patients will be described in terms of:

• Age

• Gender

• Disease duration

• Nature of cardiovascular disease:

Angina

MI

• Procedures:

Angioplasty

CABG

• Socioeconomic status (according to occupation).

### Randomised controlled trial analysis

Primary outcomes include:

• Blood pressure

• Total cholesterol

• Physical and mental status as measured by the SF-12

• Hospital re-admissions.

Secondary outcomes include:

• Measure of the process of care

SPHERE visits

Total number of GP visits

Total number of hospital OPD visits

Recording of risk factors in GP records

• Body Mass Index/Waist-Hip Ratio

• Exercise: Godin Leisure-Time Exercise Questionnaire

• Smoking status

• Diet: DINE, including question regarding portions of fresh fruit and vegetables

• Adherence to medication (Medication Adherence Report Scale MARS-5).

All results are analysed using SPSS and Stata statistical software. Statistical modelling is carried out using Stata statistical software which has a facility for complex survey sample analysis that allows for adjustments in data analysis based on design effects, planned or unplanned. Statistical significance will be taken at the 5% level for both primary and secondary outcomes. The analysis examines intervention vs control groups on study completion, as opposed to looking at changes in the intervention and control groups from baseline.

### Sub-group analyses

Sub-group analyses will be carried out on the following groups:

• Angina only vs other diagnoses

• CABG vs angioplasty

• Diabetes vs non-diabetes

• Men vs women

• Northern Ireland vs Republic of Ireland

• Patients < 70 years vs older.

Analyses will be carried out using two approaches:

• By intention to treat i.e. including all randomised patients, regardless of their participation in the intervention.

• Sensitivity analyses will explore whether adherence to the intervention influences the effect of the intervention on primary outcomes.

### Data management

The project manager has primary responsibility for the maintenance and management of the study database, which will be stored on a password-protected computer in a locked office. Research nurses have responsibility for ensuring the completeness, accuracy and confidential storage of data collected in their respective region from patient questionnaires, during the data collection consultations and from patient charts. Each practice and patient participating in the study has a unique identifying code.

Data will be collected and processed in the following ways:

1. **Questionnaire data**: once received in the practice, the research nurse collects the questionnaires and inputs the data into a master SPSS study database. Questionnaires are stored securely in their regional research centre.

2. **Consultation data**: once complete, the research nurse collects the data collection sheets from the practice and inputs the data into the master SPSS database. Data collection sheets are stored securely in their regional research centre.

3. **Chart search data**: the research nurse enters this data directly from patient notes onto an electronic study database created in Filemaker Pro software. Data can subsequently be exported directly into the master SPSS database using the unique patient identifier as the link field.

In the interests of data accuracy, numerical fields have range limiters to ensure that values outside a particular range cannot be entered; also, random data checks are performed on a regular basis to confirm completeness and accuracy of data. Inter-researcher data entry reliability is ensured by a process of trial data entry by the three research nurses of a sample of 20 questionnaires and consultation data sheets and 10 patient charts data early in the study data collection process. Where discrepancies in data entry are observed between the researchers, discussion will ensue to resolve any ambiguities or disagreement and clarify data collection and entry guidelines. Once all data has been completely entered and checked in the master SPSS database, preliminary analysis can be performed. The data will be sent in this format to the study statistician, who will import the data into Stata software to perform the necessary statistical modelling procedures.

### Data monitoring

Interim results after one year will be presented to an independent data monitoring committee (DMC), made up of a statistician, an epidemiologist, and a clinician with statistical expertise. The role of the DMC will be to:

a) Ensure that no harm is being conducted to study participants

b) Act in an advisory capacity with respect to research governance.

## Qualitative Evaluation

### Introduction

Qualitative research will be conducted after years 1 and 2 of the intervention. It will facilitate evaluation of the intervention based on the experiences of practice staff and patients in delivering and receiving the intervention respectively. It also aims to compare their experiences with those of patients and practitioners in the control practices. The qualitative research will be integrated with the economic and policy evaluations.

### Aims for intervention group (Year 1)

The aims of the qualitative evaluation study with the intervention group at the end of year 1 are to explore:

• How the intervention is implemented

• How the intervention is integrated with other practice activities

• How well practitioners and patients understand the intervention

• Whether elements of the intervention are particularly important or problematic

• Attitudes to the cost of implementing the intervention for practices and patients.

### Aims for control group (Year 1)

The aims of the qualitative evaluation study with the control group at the end of year 1 are to explore:

• The nature of secondary prevention care implemented during the study period

• The impact of any policy changes during that time.

### Aims for intervention group (Year 2)

The aims of the qualitative evaluation study with the intervention group at the end of year 2 are to explore:

• Changes in how the intervention is implemented and the reasons why

• Whether the aims of the intervention have been achieved

• The feasibility of the intervention being continued after the study ends

• Whether patients would be willing to pay for such a service.

### Aims for control group (Year 2)

The aims of the qualitative evaluation study with the control group at the end of year 2 are to explore:

• Changes in how secondary prevention care is implemented and the reasons why

• The impact of any policy changes during that time.

### Data collection and analysis

The qualitative research will be guided by the principles of the methodology of grounded theory. Practitioners and patients will be purposively sampled using theoretical and maximum variation sampling methods to ensure a variety of characteristics and experiences. Small and large practices that reflect a mixture of rural, urban, deprived and affluent locations will be selected.

The data will be collected using focus groups and individual semi-structured interviews. The focus groups aim to explore the diverse experiences of patients receiving the intervention. They will include men and women who vary in the nature of their heart disease, length of time since diagnosis, age and ethnic group (if possible). Participants will be invited to participate by letter which will explain the purpose of the interviews, followed by a telephone call from the practice nurse. Their reasons for not wishing to participate in the interviews will be recorded. The research nurses will act as observers of the focus group and will take notes of the discussion and map the interaction of the participants. Individual interviews with some patients will be used to clarify and to explore in more detail issues discussed in the focus groups. Free text comments in the patient questionnaire that forms part of the economic evaluation will also form part of the qualitative evaluation.

Individual interviews will also be conducted with GPs and practice nurses who are involved in implementing the intervention, with more interviews being conducted with the nurses, who tend to be responsible for the clinics. The interviews and focus groups will be audio-taped with the participants' consent and transcribed verbatim.

The interviews and focus groups will be divided between two qualitative researchers. The development of an interview schedule and role plays of the interviews by the researchers will be used to standardise the interviews and minimise the effects of the researchers on the data.

Data on the actual process of care will also be collected using overt observation methods by the research nurses. This will strengthen the validity of the interview data by comparing what practitioners actually do with what they say they do and will act as a quality control measure. Field notes of the research nurses' observations of the intervention based on their regular contacts with the practices will also form part of the qualitative data. These will be explored in more detail in a focus group with the research nurses.

The control practices will act as negative cases and will test whether the data from the intervention practices are unique to them or are shared with non-intervention practices. This will strengthen the reliability of the data from the intervention practices. The characteristics of the participants will be matched to the characteristics of participants in the intervention practices.

The data collection and analysis will be iterative and will continue until data saturation is achieved. Analysis will be conducted using the constant comparative method and will be facilitated by importing the interview transcripts into the computer software programme Nudist. A summary of the main findings will be sent to a sub-sample of patients and practitioners from the intervention and control groups in each of the 3 centres who were interviewed to validate our findings.

The number of interviews will be divided equally among the 3 centres. The number of interviews to be conducted among patients and practitioners is shown in Tables 1 and 2.

**Table 1:** Qualitative Research with Intervention Groups

	**Focus Groups**			**Individual Interviews**			
	
	Patient Focus Groups	Research Nurse Focus Groups	Total Focus Groups	Patient Interviews	Practice Nurse Interviews	GP Interviews	Total Interviews
Year 1	3	1	4	6	6	3	15
Year 2	3	1	4	6	6	3	15
Total	6	2	8	12	12	6	30

**Table 2:** Qualitative Research with Control Groups

	**Focus Groups**		**Individual Interviews**		
	
	Patient Focus Groups	Total Focus Groups	Practice Nurse Interviews	GP Interviews	Total Interviews
Year 1	3	3	3	3	6
Year 2	3	3	3	3	6
Total	6	6	6	6	12

## Economic Evaluation

### Introduction

The economic analysis incorporates both cost minimization analysis and cost effectiveness analysis for the various interventions for the two groups. The basic tasks of the evaluation are to identify, measure, value and compare the costs and outcomes of the alternatives being considered. Costs are likely to fall on patients, their families and the state. The health care resources consumed will reflect the costs of organising and operating the two programmes: intervention and control. These costs will reflect the time input of health professionals and fixed or overhead costs, including any new equipment and capital expenditure. All contacts with the health services are recorded and valued, including general practice contacts, hospital attendances, admissions and drug prescriptions. The patient and family costs include any out-of-pocket expenses and any own time input into the treatment process. Estimates will be made of work and leisure time foregone as part of the treatment alternatives, together with quality of life estimates. Cost-effectiveness analysis is the primary method of economic evaluation with measures of effectiveness taken from the range of primary and secondary outcomes being considered for the two groups.

In the Republic of Ireland, free healthcare is available to patients who qualify under the means-tested General Medical Scheme (GMS), although chronic disease management is not fully supported by this system. Non-GMS patients are required to pay for all of their own healthcare costs. In order that the findings of the SPHERE study will be translatable into practice within the existing healthcare systems, non-GMS patients in the Republic of Ireland will be required to pay for SPHERE-related visits to their general practice as normal. The cost of extra visits by GMS patients will be borne by the practices. The research team acknowledges that this may have an impact on recruitment of both practices and patients, although in a similar study the impact on patient recruitment was not substantial [28].

### Aim

The aim of the economic analysis is to provide both cost minimization analysis and cost-effectiveness analysis of secondary prevention of heart disease in general practice. The cost effectiveness analysis provides information on the marginal costs and effects of the intervention relative to the alternative through the calculation of an incremental cost-effectiveness ratio. In situations where there is no significant difference in effects, the use of cost minimization analysis allows the reporting of cost differences only.

### Data Collection

The following items of data are recorded to allow reporting of *health services costs*:

• Number of visits to GP in last 12 months. It was decided not to record length of consultation, but to use average length from other research in calculations.

• Number of visits to practice nurse. As length of practice nurse consultations vary, an average consultation length will be estimated by research nurses during quality control visits.

• Use of hospital services: (1) Number of attendances to outpatients in last year; (2) Number of days spent as inpatient in hospital in last year; (3) Total number of days spent in hospital in last year; (4) Number of attendances to A&E in last year.

• Current prescription of medications: type and dose, using Defined Daily Dose methodology.

• The study is not in a position to record patients' use of community services, such as physiotherapy or occupational therapy.

The following items of data are recorded to allow reporting of *patient costs*:

• Occupation data.

• Travel distance, length, mode and waiting time for visits to the practice and the hospital.

• In the Republic of Ireland, GMS status.

• Patient-estimated additional expenditure or savings made in the process of lifestyle change as a result of the intervention, e.g. changes to shopping bill with changed diet, paying for exercise facilities, giving up smoking.

The following items of data are recorded to allow reporting of *capital costs*:

• Any new equipment purchased by/for the practice to carry out the intervention.

The following items of data are recorded to allow reporting of *intervention costs*:

• All expenses associated with the intervention, such as cost of training, information and education materials.

### Outcome measures

Both primary and secondary outcome measures are collected as part of the randomised controlled trial analysis. The cost-effectiveness analysis will be based primarily on intermediate endpoints. However, some intermediate endpoints, such as blood pressure, are uni-dimensional measures and may or may not have a high correlation with a final endpoint, such as life years gained. Hence the need to explore the estimation of final endpoints in the analysis, where possible, thereby allowing much greater scope to make comparisons of cost-effectiveness with other studies.

### Analysis

The analysis will use standard economic analysis for the calculation of costs and incremental cost-effectiveness ratios. Unit costs will be applied to the resource use data to calculate the various costs of care. Comparisons between the intervention and control groups will be made on the various primary and secondary outcomes and costs will be assigned accordingly. The analysis will allow us to consider whether significant differences emerge between intervention and control groups in terms of the various outcome measures. We will examine whether the intervention is associated with overall cost increases or cost decreases and link the cost changes to incremental gains in effectiveness, where they exist.

## Policy Evaluation

A policy analysis will be carried out in the context of the economic analysis described above. There are relatively few comprehensive evaluations of health policies and even fewer that use or incorporate an experimental design as part of the appraisal process. It is expected increasingly that rigorous, replicable, relevant and independent research should be available to make a contribution to evidence-based policy [34].

A policy analysis is the process through which we identify and evaluate policies and programmes that are intended to lessen or resolve health, social, economic and physical problems relative to secondary prevention of cardiovascular disease. The proposed analysis is specific to policy circumstances in Northern Ireland and the Republic of Ireland, but its outcomes will be generalisable since these policies are examples of a wider policy movement that has affected other industrialised countries in similar ways. General conclusions can be drawn from case studies as long as data about context, processes and outcomes are collected and this study will collect such data.

The best way to assess the implications of any policy is to identify, quantify and value systematically the costs and benefits of the proposed policy. In brief, the policy appraisal process involves the following stages: defining the policy objectives; identifying the policies or programme options; identifying and measuring the costs and benefits associated with each option; identifying and assessing uncertainties; and assessing the balance between options [35].

The 5-year research programme will provide a comparative, cross-health system analytical framework with which to investigate and to evaluate the major policies relating to heart disease as articulated in *Building Healthier Hearts *[2] and in the *National Service Framework *[36], respectively. The two systems differ with respect to, for example, funding and organisational structures yet experience similar problems such as waiting lists and staff shortages. Using this framework, the policy analysis will begin by detailing and critically appraising the context, nature and distribution of heart disease in each system as well as the consequential policies and service responses. The analysis of documentary material and secondary data will be supplemented with interviews with key 'stakeholders' in the policy process. This part of the policy analysis will lead to, among other things, the identification and specification of policy goals and evaluative criteria as well as an account of the way in which the research programme will provide a 'test' and progress marker of policy objectives and policy implementation. The next step in accomplishing a thorough policy analysis is to describe and evaluate how the policy in the form of the proposed secondary prevention services in each system benefits the previously established criteria. The experimental, quantitative data relating to outcomes together with the conceptual, qualitative data about organisational and implementation processes plus the results of the economic appraisal will be analysed in an integrative way so as to evaluate and compare comprehensively the policies in each system – at least at a micro-level. This incremental, step-by-step approach plus the iterative nature of the analytical process are important because they improve the chances of producing a rigorous, context sensitive policy analysis. The research team individually and collectively will revise and deepen earlier levels of analysis as new data become available and new perspectives or interpretations emerge.

In conclusion, the comparative analytical framework will provide important insights and lessons about the process of policy development and implementation as well as about the pace and degree to which the policy in practice impacts in terms of the costs and benefits of producing "healthier hearts".

## Publications Planned

### Qualitative

• A qualitative study of the provision of secondary prevention care for established heart disease in general practice in two different health systems.

• A descriptive analysis of the development of a multidisciplinary intervention to improve secondary prevention care for established heart disease in general practice.

• A qualitative study of practitioners' experiences of a randomised controlled trial of a multidisciplinary intervention to improve secondary prevention care for established heart disease in general practice in two different health systems.

• A qualitative study of patients' responses to a multidisciplinary intervention to improve secondary prevention care for established heart disease in general practice in two different health systems.

### Main trial

• A baseline study of the provision of secondary prevention care for established heart disease in general practice in two different health systems.

• A randomised controlled trial of a multi-faceted intervention to improve secondary prevention of cardiovascular disease in general practice.

• Differences between large and small practices in a randomised controlled trial of an intervention to improve secondary prevention of cardiovascular disease in general practice.

### Economic analysis

• An economic analysis of an intervention to improve secondary prevention of heart disease in general practice.

### Policy analysis

• A policy analysis of an intervention to improve secondary prevention of heart disease in general practice in two different health systems.

### Other

• The impact of the process of care on outcomes of an intervention to improve secondary prevention of heart disease in general practice.

• The experience of implementing a randomised controlled trial to improve secondary prevention of heart disease in general practice in two different health systems.

## Competing interests

Pfizer Healthcare funds an annual teaching conference at the Department of General Practice, NUI Galway. MC is a member of Ezetrol and Inegy Primary Care Advisory Boards, funded by the Merck Sharpe Dohme /Schering Plough Partnership.

## Authors' contributions

AWM, MC, SS and MB conceived of the study and coordinated the application for funding. AWM, MC, SS, MB and CL contributed to the design of the study, drafted sections of the manuscript, and read and approved the final manuscript. SS calculated the sample size. MCB coordinated the final stages of manuscript preparation and revision.

## Supplementary Material

Additional File 1 Appendix A: Practice Recruitment Documentation Click here for file

Additional File 2 Appendix B: Patient Recruitment Documentation Click here for file

Additional File 3 Appendix C: Patient Questionnaire Click here for file

Additional File 4 Appendix D: Other Baseline Data Collection Documentation Click here for file

## References

[B1] Scottish Intercollegiate Guideline Network (2000). Secondary Prevention of Coronary Heart Disease Following Myocardial Infarction.

[B2] Cardiovascular Health Strategy Group (1999). Building Healthier Hearts.

[B3] De Backer G, Ambrosioni E, Borch-Johnsen K, Brotons C, Cifkova R, Dallongeville J (2003). European guidelines on cardiovascular disease prevention in clinical practice. European Heart Journal.

[B4] Campbell NC, Thain J, Deans HG, Ritchie LD, Rawles JM (1998). Secondary prevention in coronary heart disease: baseline survey of provision in general practice. British Medical Journal (Clinical Research Ed).

[B5] Svilaas N, Thoresen M, Kristoffersen JE, Hjartaaker J, Westheim A (2000). How well are patients with atherosclerotic disease treated? Secondary prevention in primary care. Scandinavian Journal of Primary Health Care.

[B6] Kahan T, Wandell P (2001). Risk factors in established coronary heart disease: evaluation of a secondary prevention programme. Journal of Cardiovascular Risk.

[B7] Byrne M, Murphy AW (2002). Secondary prevention of heart disease: a baseline survey of patients' lifestyles and service provision in the North Western and Western Health Boards Research and Development Report Number 2.

[B8] Connolly P, Cupples M, Cuene-Grandidier H, Johnston D, Passmore P (2002). The importance of validating the diagnosis of coronary heart disease when measuring secondary prevention: a cross sectional study in general practice. Pharmacoepidemiology and Drug Safety.

[B9] Cupples ME, McKnight A (1994). Randomised controlled trial of health promotion in general practice for patients at high cardiovascular risk. British Medical Journal.

[B10] Campbell NC, Ritchie LD, Thain J, Deans HG, Rawles JM, Squair JL (1998). Secondary prevention in coronary heart disease: a randomised trial of nurse led clinics in primary care. Heart (British Cardiac Society).

[B11] Murchie P, Campbell NC, Ritchie LD, Simpson JA, Thain J (2003). Secondary prevention clinics for coronary heart disease: four year follow up of a randomised controlled trial in primary care. British Medical Journal.

[B12] VanDrenth BB, Hulscher MEJL, Nokkink HGA, van de Lisdonk EH, van der Wouden JC, Grol RPTM (1997). Effects of outreach visits by trained nurses on cardiovascular risk factor recording in general practice. European Journal of General Practice.

[B13] Jolly K, Bradley F, Sharp S (1999). Randomised controlled trial of follow up care in general practice of patients with myocardial infarction and angina: final results of the Southampton heart integrated care project (SHIP). The SHIP Collaborative Group. British Medical Journal.

[B14] Feder G, Griffiths C, Eldridge S, Spence M (1999). Effect of postal prompts to patients and general practitioners on the quality of primary care after a coronary event (POST): randomised controlled trial. British Medical Journal.

[B15] Moher M, Yudkin P, Wright L (2001). Cluster randomised controlled trial to compare three methods of promoting secondary prevention of coronary heart disease in primary care. British Medical Journal.

[B16] Langham J, Tucker H, Sloan D, Pettifer J, Thom S, Hemingway H (2002). Secondary prevention of cardiovascular disease: a randomised controlled trial of training in information management, evidence-based medicine, both or neither: the PIER trial. British Journal of General Practice.

[B17] Lewin RJP, Furze G, Robinson J (2002). A randomised controlled trial of a self-management plan for patients with newly diagnosed angina. British Journal of General Practice.

[B18] McAlister FA, Lawson FME, Teo KK, Armstrong PW (2001). Randomised trials of secondary prevention programmes in coronary heart disease: Systematic review. British Medical Journal.

[B19] Ebrahim S, Smith GD (1997). Systematic review of randomised controlled trials of multiple risk factor interventions for preventing coronary heart disease. British Medical Journal.

[B20] Wiles R (1998). Patients' perceptions of their heart attack and recovery: the influence of epidemiological "evidence" and personal experience. Social Science and Medicine.

[B21] Begg A (2003). Tackling the clinical indicators: secondary prevention of CHD. Guidelines in Practice.

[B22] Steptoe A, Doherty S, Rink E, Kerry S, Kendrick T, Hilton S (1999). Behavioural counselling in general practice for the promotion of healthy behaviour among adults at increased risk of coronary heart disease: randomised trial. British Medical Journal.

[B23] Steptoe A, Perkins-Porras L, McKay C, Rink E, Hilton S, Cappucio FP (2003). Behavioural counselling to increase consumption of fruit and vegetables in low income adults: randomised trial. British Medical Journal.

[B24] Hulscher M, Wensing M, van der Weijen T, Grol R (2001). Interventions to implement prevention in primary care (Cochrane review). The Cochrane Library.

[B25] Cupples ME, McKnight A (1999). Five year follow up of patients at high cardiovascular risk who took part in a randomised controlled trial of health promotion. British Medical Journal.

[B26] Stewart M (1995). Effective physician-patient communication and health outcomes: a review. Canadian Medical Association Journal.

[B27] Kinmonth AL, Woodcock A, Griffin S, Spiegal N, Campbell MJ (1998). Randomised controlled trial of patient centred care of diabetes in general practice: impact on current wellbeing and future disease risk. The Diabetes Care From Diagnosis Research Team. British Medical Journal.

[B28] Smith S, Byrne G, Shannon WF, Thompson C, O'Leary M, Staines A, Tynan A (2001). The North Dublin Diabetes Shared Care (DiSC) Project: A profile of current diabetes care in Ireland. Irish Medical Journal.

[B29] Ware JE (1993). SF-36 health survey – manual and interpretation guide.

[B30] Emmons KM, Rollnick S (2001). Motivational interviewing in health care settings: Opportunities and limitations. American Journal of Preventive Medicine.

[B31] Rollnick S, Kinnersley P, Stott N (1993). Methods of helping patients with behaviour change. British Medical Journal.

[B32] Bandura A (1977). Social Learning Theory.

[B33] Kelleher C, Nic Gabhainn S, Friel S, Corrigan H, Nolan G, Sixsmith J (2003). The National Health and Lifestyle Surveys.

[B34] Economic and Social Research Council Centre for Evidence Based Policy and Practice. http://www.evidencenetwork.org.

[B35] Policy Planning Research Unit (1996). Policy Evaluation: Occasional Paper no 32.

[B36] National Health Service (2000). National Service Framework for Coronary Heart Disease.

