# Supplementary material for: The SPHERE Study. Secondary prevention of heart disease in general practice: protocol of a randomised controlled trial of tailored practice and patient care plans with parallel qualitative, economic and policy analyses. [ISRCTN24081411]
Source: Curr Control Trials Cardiovasc Med. 2005 Jul 29;6(1):11. doi: 10.1186/1468-6708-6-11 (PMC1208929; doi:10.1186/1468-6708-6-11)
Supplement: Additional File 1 — Appendix A: Practice Recruitment Documentation [file 1468-6708-6-11-S1.doc]

APPENDIX A: PRACTICE RERUITMENT DOCUMENTATION

PRACTICE INVITATION LETTER

PRACTICE INFORMATION SHEET (REPUBLIC OF IRELAND)

PRACTICE INFORMATION SHEET (NORTHERN IRELAND)

PRACTICE IMPLEMENTATION PLAN

INFORMATION TO PRACTICES REGARDING RANDOM SELECTION OF PATIENTS

PRACTICE INVITATION LETTER

Invite letter recipient

Practice Address 1

Address 2

Address 3

Address 4

XX.XX.XX

Dear <Invite letter recipient>

Many thanks for taking the time to speak with our Research Nurse <AH/CL/MOM> on <Date> about the SPHERE Study. Further to your interest in learning more about the study, please find enclosed an information sheet which provides more detail about SPHERE.

<Ailish/Claire/Mary> will phone you again during the next ten days to answer any queries you may have about the study. If you wish to meet her to discuss any issues further, or if you are interested in participating, she will arrange a time for a meeting in your practice. This visit will be brief and will be arranged at a time chosen by you. Lunch will be provided if this visit is arranged during lunch hour. All staff are welcome to attend.

In the meantime, I would be grateful if you could circulate the enclosed information to all staff in your practice. Please call < AH/CL/MOM > on <……….> or myself on <………..> if you would like to discuss any matters relating to the study.

Alternatively if you do not wish to participate, we thank you for taking the time to read the enclosed information.

With best wishes,

__________________

Mary Byrne

SPHERE Study Manager

PRACTICE INFORMATION SHEET – Republic of Ireland

|  | **The SPHERE Study** **Secondary Prevention of Heart DiseasE in GeneRal PracticE** ***A research study funded by the Health Research Board*** |
| --- | --- |

**Your Project Contacts**

Name:XXX Name: XXX

Role: Research Nurse Role: Project Manager

Phone: XXX Phone: XXX

Mobile: XXX Mobile: XXX

E-mail: XXX E-mail: XXX

Available: Monday - Friday, 09.15 - 17.15 Available: Monday – Friday, 09.15 - 17.15

### PRACTICE INFORMATION SHEET

***Project Title***

A randomised controlled trial of a tailored intervention to improve secondary prevention of coronary heart disease in general practice, based on the principles of patient-centred care and the most recent research findings.

***Short title***

The SPHERE Study (Secondary Prevention of Heart DiseasE in GeneRal PracticE).

***Purpose***

To assess the benefit of additional training and support to practices in reducing risk for patients who have been diagnosed with coronary heart disease (CHD). The intervention will aim to facilitate practitioners in secondary prevention, specifically to:

• Increase appropriate medication prescribing and patient medication adherence

• Help patients to adopt healthier lifestyles.

***Evaluation***

The SPHERE study intervention will be evaluated by randomised controlled trial, involving both intervention and control practices. Randomisation will take place following baseline data collection from all eligible patients.

***Host Institutions***

National University of Ireland, Galway (Department of General Practice)

Queen’s University Belfast (Department of General Practice)

Trinity College Dublin (Department of Public Health and Primary Care)

***Researchers***

Professor Andrew Murphy, Ms. Ailish Houlihan, Ms. Mary Byrne, Dr. Molly Byrne.

Dr. Margaret Cupples, Ms. Claire Leathem, Dr. Mairead Corrigan.

Dr. Susan Smith, Ms. Mary O Malley.

***Funder***

The Health Research Board.

***What will happen if my practice agrees to participate in SPHERE?***

- A Research Nurse will visit your practice for approximately 1.5hrs to give information regarding the study’s aims and objectives and to answer any questions.
- All staff will be welcomed to indicate their commitment by signing a practice staff consent form.
- Practice staff will agree upon a key person (GP or Practice Manager) for further contact regarding visit dates.
- The Research Nurse will facilitate each practice in establishing a register of patients with established Coronary Heart Disease (i.e. documented MI, angina, angioplasty or CABG) from their patient lists.
- Twenty randomly selected patients from this list will be invited to participate in the study.
- Interested patients will receive a letter from their GP, a SPHERE information leaflet and a postal questionnaire. They will be invited to attend their practice for a baseline consultation with their GP/Practice Nurse, during which clinical measurements of blood pressure and cholesterol will be recorded and medication reviewed.
- During this first consultation the patient will be invited to fill out a consent form indicating their willingness to participate in SPHERE and permitting the Research Nurse to take patient data from their medical notes.
- Practices and patients will be informed that they have an equal chance of being selected for the intervention or control arm of the study.

**Baseline Data Collection involves:**

| Patient questionnaire | Each patient will receive a postal questionnaire. |
| --- | --- |
| Data from Patient Consultation | Data (BP, cholesterol, etc.) will be collected by the Practice Nurse/GP and recorded on a two-sheet data collection form. |
| Patient Records | Charts of all participating patients will be searched by the SPHERE Research Nurse. |

**At this point, participating practices will be randomly assigned to**

## **Intervention or Control (usual care) practices**

***What will happen if my practice is assigned to be an Intervention Practice?***

- An intervention meeting with practice staff will be arranged by the Research Nurse, in conjunction with the key GP/Practice Manager. This meeting will last no longer than 1.5 hours and will be held in the practice. During the meeting, further detail about the intervention will be provided, intervention materials will be disseminated, and current medical guidelines in secondary prevention of CHD will be provided and discussed.
- The needs of your practice will be assessed and a tailored practice plan will be devised describing how the SPHERE intervention will be implemented in the practice.
- Practice staff will be invited to attend one further training session which will be based on lifestyle behaviour change. Again, this training session will last no longer than 1.5 hours and will be held in the practice. The content of this session will be determined by the needs expressed by practice staff.
- The ICGP has granted approval for GMS study leave and competence assurance accreditation for SPHERE training.

**The Intervention Process involves:**

Consultations Each SPHERE patient will see their GP or Practice Nurse every four

months for a consultation with a special emphasis on behaviour

change and secondary prevention medications. Clinical assessment

(BP, cholesterol, etc.) will take place at year 1 and year 2.

Data Collection Patient postal questionnaire and chart search at year 1 and year 2.

***What will happen if my practice is assigned to be a Control Practice?***

Patients will continue to receive usual care from their GP/Practice Nurse.

**The Control Process involves:**

Consultations Usual Care.

Each SPHERE patient will be invited to attend a consultation with

their Practice Nurse at year 1 and year 2 for a clinical assessment

(BP, cholesterol, etc.).

Data Collection Patient postal questionnaire and chart search at year 1 and year 2.

***Some General Information for both Intervention and Control practices . . .***

**Is there any payment?**

YES, both Intervention and Control practices will receive an honorarium of €1000 on completion of the study.

**Has Ethical Approval been granted?**

YES, ethical approval has been granted by the Ethics Committee of the Irish College of General Practitioners and the Research Ethics Committee of Queen’s University, Belfast.

**How long will the study last?**

The study will take place over a two-year period, with data collection at baseline, 1 year and 2 years. The Research Nurse will offer support and assistance to practice staff participating in SPHERE throughout the duration of the study.

**Quality Control**

The Research Nurse may observe some consultations between patient and GP or Practice Nurse, to assess whether the training has had an impact on practice.

###### Consent and Confidentiality

- All patients selected to participate in the study will receive detailed information and sign a consent form before taking part.
- Patients declining to participate in the study will be reassured that their care will continue on as previously.
- All patients taking part in the study will be assigned a study ID number, so their data will be stored anonymously. All individual patient data will be stored securely and only accessed by a member of the research team.

*We thank you for your help and interest in this project.*

*If you would like any further information or clarification, please call*

*Mary Byrne, Project Manager on 091-495205*

*or e-mail* [*mary.byrne@nuigalway.ie*](mailto:molly.byrne@nuigalway.ie)

PRACTICE INFORMATION SHEET – Northern Ireland

|  | **The SPHERE Study** **Secondary Prevention of Heart DiseasE in GeneRal PracticE** *A research study funded by the Health Research Board* |
| --- | --- |

**Your Project Contacts**

Name:XXX Name: XXX

Role: Research Nurse Role: Project Manager

Phone: XXX Phone: XXX

Mobile: XXX Mobile: XXX

E-mail: [XXX](mailto:c.leathem@qub.ac.uk) E-mail: XXX

Available: Monday - Friday, 09.15 - 17.15 Available: Monday – Friday, 09.15 - 17.15

### PRACTICE INFORMATION SHEET

***Project Title***

A randomised controlled trial of a tailored intervention to improve secondary prevention of coronary heart disease in general practice, based on the principles of patient-centred care and the most recent research findings.

***Short title***

The SPHERE Study (Secondary Prevention of Heart DiseasE in GeneRal PracticE).

***Purpose***

To assess the benefit of additional training and support to practices in reducing risk for patients who have been diagnosed with coronary heart disease (CHD). The intervention will aim to facilitate practitioners in secondary prevention, specifically to:

• Increase appropriate medication prescribing and patient medication adherence

• Help patients to adopt healthier lifestyles.

***Evaluation***

The SPHERE Study intervention will be evaluated by randomised controlled trial, involving both intervention and control practices. Randomisation will take place following baseline data collection from all eligible patients.

***Host Institutions***

Queen’s University Belfast (Department of General Practice)

National University of Ireland, Galway (Department of General Practice)

Trinity College Dublin (Department of Public Health and Primary Care)

***Researchers***

Dr. Margaret Cupples, Ms. Claire Leathem, Dr. Mairead Corrigan.

Professor Andrew Murphy, Ms. Ailish Houlihan, Ms. Mary Byrne, Dr. Molly Byrne.

Dr. Susan Smith, Ms. Mary O Malley.

***Funder***

The Health Research Board.

***What will happen if my practice agrees to participate in SPHERE?***

- A Research Nurse will visit your practice for a short meeting to give information regarding the study’s aims and objectives and to answer any questions.
- All staff will be welcomed to indicate their commitment by signing a practice staff consent form.
- Practice staff will agree upon a key person (GP or Practice Manager) for further contact regarding visit dates.
- The Research Nurse will facilitate each practice in establishing a register of patients with established Coronary Heart Disease (i.e. documented MI, angina, angioplasty or CABG) from their patient lists.
- Twenty randomly selected patients from this list will be invited to participate in the study.
- Interested patients will receive a letter from their GP, a SPHERE information leaflet and a postal questionnaire. They will be invited to attend their practice for a baseline consultation with their GP/Practice Nurse, during which clinical measurements of blood pressure and cholesterol will be recorded and medication reviewed.
- During this first consultation the patient will be invited to fill out a consent form indicating their willingness to participate in SPHERE and permitting the Research Nurse to take patient data from their medical notes.
- Practices and patients will be informed that they have an equal chance of being selected for the intervention or control arm of the study.

**Baseline Data Collection involves:**

| Patient questionnaire | Each patient will receive a postal questionnaire. |
| --- | --- |
| Data from Patient Consultation | Data (BP, cholesterol, etc.) will be collected by the Practice Nurse/GP and recorded on a one-page data collection form. |
| Patient Records | Charts of all participating patients will be searched by the SPHERE Research Nurse. |

**At this point, participating practices will be randomly assigned to**

## **Intervention or Control (usual care) practices**

***What will happen if my practice is assigned to be an Intervention Practice?***

- An intervention meeting with practice staff will be arranged by the Research Nurse, in conjunction with the key GP/Practice Manager. This meeting will last no longer than 1.5 hours and will be held in the practice. During the meeting, further detail about the intervention will be provided, intervention materials will be disseminated, and current medical guidelines in secondary prevention of CHD will be provided and discussed.
- The needs of your practice will be assessed and a tailored practice plan will be devised describing how the SPHERE intervention will be implemented in the practice.
- Practice staff will be invited to attend one further training session which will be based on lifestyle behaviour change. Again, this training session will last no longer than 1.5 hours and will be held in the practice. The content of this session will be determined by the needs expressed by practice staff.
- Practitioners’ participation in SPHERE training sessions has been approved by the Northern Ireland Medical and Dental Training Agency (NIMDTA) as an accredited contribution to their annual appraisal.

**The Intervention Process involves:**

Consultations Each SPHERE patient will see their GP or Practice Nurse every four

months for a consultation with a special emphasis on behaviour

change and secondary prevention medications. Clinical assessment

(BP, cholesterol, etc.) will take place at year 1 and year 2.

Data Collection Patient postal questionnaire and chart search at year 1 and year 2.

***What will happen if my practice is assigned to be a Control Practice?***

Patients will continue to receive usual care from their GP/Practice Nurse.

**The Control Process involves:**

Consultations Usual Care.

Each SPHERE patient will be invited to attend a consultation with

their Practice Nurse at year 1 and year 2 for a clinical assessment

(BP, cholesterol, etc.).

Data Collection Patient postal questionnaire and chart search at year 1 and year 2.

***Some General Information for both Intervention and Control practices . . .***

**Is there any payment?**

YES, both Intervention and Control practices will receive an honorarium of £650 on completion of the study.

**Has Ethical Approval been granted?**

YES, ethical approval has been granted by the Research Ethics Committee of Queen’s University, Belfast and the Ethics Committee of the Irish College of General Practitioners.

**How long will the study last?**

The study will take place over a two-year period, with data collection at baseline, 1 year and 2 years. The Research Nurse will offer support and assistance to practice staff participating in SPHERE throughout the duration of the study.

**Quality Control**

The Research Nurse may observe some consultations between patient and GP or Practice Nurse, to assess whether the training has had an impact on practice.

###### Consent and Confidentiality

- All patients selected to participate in the study will receive detailed information and sign a consent form before taking part.
- Patients declining to participate in the study will be reassured that their care will continue on as previously.
- All patients taking part in the study will be assigned a study ID number, so their data will be stored anonymously. All individual patient data will be stored securely and only accessed by a member of the research team.

*We thank you for your help and interest in this project.*

*If you would like any further information or clarification, please call*

*Mary Byrne, Project Manager on +353-91-495205*

*or e-mail* [*mary.byrne@nuigalway.ie*](mailto:molly.byrne@nuigalway.ie)

PRACTICE IMPLEMENTATION PLAN

# SPHERE

Secondary Prevention of Heart DiseasE in GeneRal PracticE

PRACTICE IMPLEMENTATION PLAN

This form should be completed at the first meeting between the practice and the SPHERE research team. It can be kept as a record of who is responsible within your practice for the different tasks in the study.

**Practice Name____________________­­­_____ Practice Number_________**

| Aim | Tasks | Name(s) |
| --- | --- | --- |
| 1. Randomly select 20 patients from current CHD list for participation in SPHERE. | - Assign a study number to all CHD patients (from 1 to x). - Use computer generated random numbers (provided by SPHERE team) to select 20 patients from the list. | _____________________  (and SPHERE Research Nurse) |
| 1. Approach eligible patients re SPHERE. | - Post out patient letter, information sheet, questionnaire and reply slip. - Receive returned letters.  1. *Patient interested:*   Contact for an appointment.   1. *Patient not interested:*   Do not contact.   1. *Non responder:*   Send reminder letter and/or phone. | _______________________  _______________________ |
| 1. Confirm patient participation in SPHERE study at   first consultation. | - Explain study and obtain patient’s informed consent. - Record Baseline data. | _______________________  (Practice Nurse / GP)  _______________________  (Practice Nurse / GP) |

INFORMATION TO PRACTICES REGARDING RANDOM SELECTION OF PATIENTS

|  | **The SPHERE Study** **Secondary Prevention of Heart DiseasE in GeneRal PracticE** ***A research study funded by the Health Research Board*** |
| --- | --- |

**Information to Practices**

**regarding**

### Random Selection of Patients

Many thanks for your participation with the SPHERE study to date.

The next stage of the study involves the selection of patients from your practice who will participate in the study. Patients need to be selected in a particular way to ensure that they are a random sample of your practice. We would like to have 20 patients from your practice participating in the study.

Patients who are selected will be sent an invitation letter from the practice, with an information sheet about the study and a questionnaire about health-related aspects of their lifestyle. They will be asked to complete the questionnaire and a reply slip indicating their interest in participating, and to return these to the practice in a pre-stamped, pre-addressed envelope. Your regional SPHERE research nurse will be in touch with you to assist with sending these letters to 25 patients from your list of eligible patients, in the hope that this will guarantee 20 patients for the study.

The research nurse will work with you to follow the steps below to select patients from the list you have already generated in your practice:

Step 1. On the list you have already generated of all potential patients, each patient will be allocated a number: the first patient will be given number 1, the second number 2, etc. until all patients have a number.

Step 2. Another list of numbers arranged in random order will be held at a separate location until your practice’s patients have had their numbers allocated.

Step 3. The first 25 numbers in this random order will be used to select the patients who will be sent letters inviting them to take part in the study. If at least 6 of the invited patients do not agree to take part, the patient corresponding to the next number on the random numbers list will be invited until the quota of 20 participating patients is reached. Please keep this list of patients with allocated numbers carefully, in case you may need to refer to it again.

If you have any questions whatsoever regarding selecting patients please do not hesitate to contact Mary Byrne, Project Manager on 091-495205 (from the Republic of Ireland) or +353-91-495205 (from the North of Ireland), or your regional Research Nurse.
