# Supplementary material for: The SPHERE Study. Secondary prevention of heart disease in general practice: protocol of a randomised controlled trial of tailored practice and patient care plans with parallel qualitative, economic and policy analyses. [ISRCTN24081411]
Source: Curr Control Trials Cardiovasc Med. 2005 Jul 29;6(1):11. doi: 10.1186/1468-6708-6-11 (PMC1208929; doi:10.1186/1468-6708-6-11)
Supplement: Additional File 2 — Appendix B: Patient Recruitment Documentation [file 1468-6708-6-11-S2.doc]

APPENDIX B: PATIENT RECRUITMENT DOCUMENTATION

PATIENT INVITATION LETTER

PATIENT INFORMATION SHEET (REPUBLIC OF IRELAND)

PATIENT INFORMATION SHEET (NORTHERN IRELAND)

PATIENT REPLY SLIP

PATIENT CONSENT FORM

PATIENT INVITATION LETTER

<Practice Name>

<Practice Address>

<Line 2>

<Line 3>

<Postcode>

<Telephone Number>

<Date>

Dear <Insert Patient Name>

Our practice has agreed to help with a study looking into improving care for patients with coronary heart disease. We would like to invite you to take part in this study.

We have enclosed an information sheet that describes the study in more detail. We hope that it will answer some of the questions you may have about taking part. However if you would like to ask any other questions about the research, please feel free to get in touch with <Insert name> at the surgery, who will be happy to answer your questions.

If you are interested in taking part, please complete the reply slip and the questionnaire enclosed and return them in the envelope provided. It would be very helpful if you could return the completed questionnaire even if you do not want to take part in the study.

While your help in this project would be greatly appreciated, taking part is completely voluntary. If you do not want to take part, it will not affect the care you receive from the surgery in any way.

Many thanks for your help.

Yours sincerely

< GP Signature>

PATIENT INFORMATION SHEET (Republic of Ireland)

|  | **The SPHERE Study** **Secondary Prevention of Heart DiseasE in GeneRal PracticE** *A research study funded by the Health Research Board* |
| --- | --- |

**PATIENT INFORMATION SHEET**

**What is the SPHERE study?**

The aim of the SPHERE study is to determine whether a specific management programme for patients with heart disease is better than the care provided for these patients currently. This study has been funded by the Health Research Board, and is being carried out by researchers from NUI Galway, Trinity College Dublin and Queen’s University Belfast.

General practices participating in the study will be divided randomly (that is, by chance) into 2 groups: Some of the practices will continue to care for their patients in the usual way and others will be given specific help in providing advice and treatment plans to patients. Towards the end of the study, which is planned to last for two years, the specific programme provided by the research team will be offered to all practices.

Why have I been invited to participate?

The general practice which you attend has agreed to participate in this study. Patients with heart disease in your practice are being invited to take part.

**What will happen if I agree to participate in the study?**

1. A researcher from the study will record some basic information about your condition from your medical charts. This data will be treated confidentially and recorded anonymously.

2. You will be invited to attend your GP or practice nurse for a basic health assessment, which will involve the following:

- Your weight, height and blood pressure will be measured.
- A small sample of blood will be taken to measure your cholesterol level (if this has not been done recently).
- Your medication will be reviewed.

3. If your practice is chosen to provide a specific management programme, you will be invited to attend the surgery following your health assessment to collect your results and review your current treatment. After this you will be invited to attend the practice every four months for two years. At the end of the first and second years of the study, you will be invited to attend for a repeat of the basic health assessment described above.

4. If your practice has **not** been selected for a specific management programme, your usual care will continue to be provided. You will be invited to attend the surgery after one year and after two years for a repeat of the basic health assessment described above.

5. During the course of the study, **a small number of patients** will be randomly (that is, by chance) invited to take part in group discussions regarding their experience of the management of heart disease. If you are selected for such an invitation you will be totally free to refuse to participate in these discussions or to withdraw from them at any time without causing displeasure and without affecting your future medical or nursing care.

**What will happen if I don’t participate in the study?**

Your participation in SPHERE is completely voluntary. If you decide not to take part, or if you later decide to withdraw from the study at any time:

- You may do so freely without causing displeasure.
- You do not have to give a reason.
- Your future treatment will not be affected in any way.

**Will I have to pay?**

If you do not have a medical card, you will be asked to pay for your visits to the surgery in the usual way. If you have a medical card, you will not be asked to pay.

**Will my privacy be protected?**

In any report or publication of the study findings you will not be identified. All information relevant to you will be treated confidentially and recorded anonymously.

**What should I do now?**

If you would like to participate in the study, please:

- Complete the enclosed reply slip
- Complete the enclosed questionnaire
- Return both to your practice in the stamped, addressed envelope provided.

If you would like some help completing the questionnaire, please do not hesitate to contact someone from the research team who will be pleased to assist you (contact numbers at the end of this leaflet).

If you would **not** like to participate in the study, please complete the enclosed reply slip and return it to your practice in the stamped, addressed envelope provided.

**Where can I get more information?**

If you would like more information about the study, or if you would like some help completing the enclosed questionnaire, please contact one of the following people:

Mary Byrne Mary O’Malley

Project Manager Regional Research Nurse

Department of General Practice Dept. of Public Health & Primary Care

1 Distillery Road Trinity College Centre for Health Sciences

NUI Galway Adelaide & Meath Hospital, Tallaght, D.24

Tel: 091 – 495205 Tel: 01 - 6081545

Email: [mary.byrne@nuigalway.ie](mailto:mary.byrne@nuigalway.ie) Email: momalle@tcd.ie

## **Thank you for taking the time to read this information sheet!**

PATIENT INFORMATION SHEET (Northern Ireland)

|  | **The SPHERE Study** **Secondary Prevention of Heart DiseasE in GeneRal PracticE** *A research study funded by the Health Research Board* |
| --- | --- |

**PATIENT INFORMATION SHEET**

**What is the SPHERE study?**

The aim of the SPHERE study is to determine whether a specific management programme for patients with heart disease is better than the care provided for these patients currently. This study has been funded by the Health Research Board, and is being carried out by researchers from NUI Galway, Trinity College Dublin and Queen’s University Belfast.

General practices participating in the study will be divided randomly (that is, by chance) into 2 groups: Some of the practices will continue to care for their patients in the usual way and others will be given specific help in providing advice and treatment plans to patients. Towards the end of the study, which is planned to last for two years, the specific programme provided by the research team will be offered to all practices.

Why have I been invited to participate?

The general practice which you attend has agreed to participate in this study. Patients with heart disease in your practice are being invited to take part.

**What will happen if I agree to participate in the study?**

1. A researcher from the study will record some basic information about your condition from your medical charts. This data will be treated confidentially and recorded anonymously.

2. You will be invited to attend your GP or practice nurse for a basic health assessment, which will involve the following:

- Your weight, height and blood pressure will be measured.
- A small sample of blood will be taken to measure your cholesterol level (if this has not been done recently).
- Your medication will be reviewed.

3. If your practice is chosen to provide a specific management programme, you will be invited to attend the surgery following your health assessment to collect your results and review your current treatment. After this you will be invited to attend the practice every four months for two years. At the end of the first and second years of the study, you will be invited to attend for a repeat of the basic health assessment described above.

4. If your practice has **not** been selected for a specific management programme, your usual care will continue to be provided. You will be invited to attend the surgery after one year and after two years for a repeat of the basic health assessment described above.

5. During the course of the study, **a small number of patients** will be randomly (that is, by chance) invited to take part in group discussions regarding their experience of the management of heart disease. If you are selected for such an invitation you will be totally free to refuse to participate in these discussions or to withdraw from them at any time without causing displeasure and without affecting your future medical or nursing care.

**What will happen if I don’t participate in the study?**

Your participation in SPHERE is completely voluntary. If you decide not to take part, or if you later decide to withdraw from the study at any time:

- You may do so freely without causing displeasure.
- You do not have to give a reason.
- Your future treatment will not be affected in any way.

**Will my privacy be protected?**

In any report or publication of the study findings you will not be identified. All information relevant to you will be treated confidentially and recorded anonymously.

**What should I do now?**

If you would like to participate in the study, please:

- Complete the enclosed reply slip
- Complete the enclosed questionnaire
- Return both to your practice in the stamped, addressed envelope provided.

If you would like some help completing the questionnaire, please do not hesitate to contact someone from the research team who will be pleased to assist you (contact numbers at the end of this leaflet).

If you would **not** like to participate in the study, please complete the enclosed reply slip and return it to your practice in the stamped, addressed envelope provided.

**Where can I get more information?**

If you would like more information about the study, or if you would like some help completing the enclosed questionnaire, please contact one of the following people:

Mary Byrne Claire Leathem

Project Manager Principal Research Nurse

Department of General Practice Department of General Practice

1 Distillery Road Queen’s University Belfast

NUI Galway Dunluce Health Centre, Belfast BT9 7HR

Tel: 091 - 495205 Tel: 028 - 90204340

Email: [mary.byrne@nuigalway.ie](mailto:mary.byrne@nuigalway.ie) Email: [c.leathem@qub.ac.uk](mailto:c.leathem@qub.ac.uk)

## **Thank you for taking the time to read this information sheet!**

PATIENT REPLY SLIP

|  | **The SPHERE Study** **Secondary Prevention of Heart DiseasE in GeneRal PracticE** *A research study funded by the Health Research Board* |
| --- | --- |

**Patient Reply Slip**

*Please return this reply slip to your practice, in the envelope provided.*

My name is ___________________________

| **I am interested** in taking part in this study and I would like to hear more | Please tick one of the boxes |
| --- | --- |
| I would **not** like to take part in this study |  |

- If you are interested in this study and would like to hear more, someone from the practice will ring you when you have returned this reply sheet. They will answer any questions you may have and confirm the date for your next appointment at the surgery. Please write your telephone number below.
- If you would not like to take part in this study, you will not hear from the practice again about the study.

**My telephone number is ___________________**

*Many thanks. Your help with this study is appreciated.*

PATIENT CONSENT FORM

|  | **The SPHERE Study** **Secondary Prevention of Heart DiseasE in GeneRal PracticE** *A research study funded by the Health Research Board* |
| --- | --- |

**PATIENT CONSENT FORM**

Patient Name ___________________________

Date of Birth ______/______/_______

Study ID Number __________________

The SPHERE study has been explained to me

and I have been given the opportunity to ask Yes  No 

further questions.

I understand that a researcher from the SPHERE

study will record some basic information about Yes  No 

my condition from my medical charts.

I understand that my participation in this study is

voluntary and that I am free to refuse or withdraw Yes  No 

at any time without causing displeasure or affecting

my future medical or nursing care.

I agree to be contacted by a SPHERE researcher after

one year, and again after two years, with a request Yes  No 

for follow-up information.

I agree to take part in the study. Yes  No 

Patient Signature _____________________________ Date ____________

*Witnessed by:*

Nurse/GP Signature __________________________ Date ____________

Nurse/GP Name (block capitals) __________________________________
