# Supplementary material for: The SPHERE Study. Secondary prevention of heart disease in general practice: protocol of a randomised controlled trial of tailored practice and patient care plans with parallel qualitative, economic and policy analyses. [ISRCTN24081411]
Source: Curr Control Trials Cardiovasc Med. 2005 Jul 29;6(1):11. doi: 10.1186/1468-6708-6-11 (PMC1208929; doi:10.1186/1468-6708-6-11)

APPENDIX C: PATIENT QUESTIONNAIRE

**ID:**

###

Secondary Prevention of Heart DiseasE in GeneRal PracticE

**Patient**

###### Questionnaire

If you are interested in taking part in the SPHERE study,

please fill out this questionnaire.

It will take about 30 minutes to complete.

The questionnaire has 6 sections: A,B,C,D,E and F.

Please try to answer ALL the questions.

If you would like some help answering the questions, please phone one of the following numbers (9.30am–5pm) and we will be pleased to assist you:

- Northern Ireland: Tel. 028-90204340 / 077-10030399
- Dublin/Wicklow/Kildare: Tel. 01-6081545 / 087-2490113

##### West/North West: Tel. 091-495205 / 087-2490113

##### When you have finished the questionnaire, please post it back to your GP surgery using the Stamped Addressed Envelope provided.

##### Thank you very much for your help!

Section A: Your General Health

*This part of the survey asks for your views about your health.*

*Answer every question by marking the answer as indicated.*

***For example****, in question A1 if you want to say that your health is good,*

*you would tick the box beside ‘good’.*

*If you are unsure about how to answer a question,*

*please give the best answer you can.*

**A1. In general, would you say your health is:**

Excellent ****

Very Good ****

Good ****

Fair ****

Poor ****

**A2.** The following questions are about things you might do during a typical day. Does your health now limit you in these things? If so, how much?

**Moderate activities, such as moving a table, vacuum cleaning, bowling or playing golf**

Yes, limited a lot ****

Yes, limited a little ****

No, not limited at all ****

**Climbing several flights of stairs**

Yes, limited a lot ****

Yes, limited a little ****

No, not limited at all ****

**A3.** During the past 4 weeks, how much of the time have you had any of the following problems with your work or other regular daily activities as a result of your physical health?

**I have been able to do less than I would like**

All of the time ****

Most of the time ****

Some of the time ****

A little of the time ****

None of the time ****

**I was limited in the kind of work or other activities**

All of the time ****

Most of the time ****

Some of the time ****

A little of the time ****

None of the time ****

**A4.** During the past 4 weeks, how much of the time have you had any of the following problems with your work or other regular daily activities as a result of any emotional problems (such as feeling depressed or anxious)?

**I have been able to do less than I would like**

All of the time ****

Most of the time ****

Some of the time ****

A little of the time ****

None of the time ****

**I did things less carefully than usual**

All of the time ****

Most of the time ****

Some of the time ****

A little of the time ****

None of the time ****

**A5.** During the past 4 weeks, how much did pain interfere with your normal work (including both work outside the home and housework)?

**** Not at all **** A little bit **** Moderately **** Quite a bit **** Extremely

**A6.** These questions are about how you feel and how things have been with you during the past 4 weeks. For each question, please give the one answer that comes closest to the way you have been feeling.

How much of the time during the past 4 weeks.....

#### Have you felt peaceful and calm?

All of the time ****

Most of the time ****

Some of the time ****

A little of the time ****

None of the time ****

**Did you have a lot of energy?**

All of the time ****

Most of the time ****

Some of the time ****

A little of the time ****

None of the time ****

**Have you felt downhearted and depressed?**

All of the time ****

Most of the time ****

Some of the time ****

A little of the time ****

None of the time ****

**A7.** During the past 4 weeks, how much of the time has your physical health or emotional problems interfered with your normal social activities (like visiting with family and friends)?

All of the time ****

Most of the time ****

Some of the time ****

A little of the time ****

None of the time ****

Section B: Exercise

*This section is about your leisure activity and how much exercise you take.*

**B1. In a normal week, how many times on average do you do the following kinds of exercise for more than 15 minutes during your free time?**

*(Please write the number of times on* ***each*** *line)*

|  | Times per Week |
| --- | --- |

| **a) STRENUOUS EXERCISE (HEART BEATS RAPIDLY)** |  |
| --- | --- |
| (e.g. running, jogging, hockey, football, soccer,  squash, basketball, judo, roller skating,  vigorous swimming, vigorous long distance cycling) | _______ |
| **b) MODERATE EXERCISE (NOT EXHAUSTING)** |  |
| (e.g. fast walking, tennis, badminton,  easy swimming, easy cycling, volleyball,  baseball, dancing, heavy gardening) | _______ |
| **c) MILD EXERCISE (MINIMAL EFFORT)** |  |
| (e.g. yoga, golf, easy walking, fishing,  bowling, light gardening) | _______ |

**B2. In a typical week, during your leisure time, how often do you engage in any regular activity, such as jogging or cycling, long enough to work up sweat?**

*(Please tick* ✓ *the best box for you)*

Often ****

Sometimes ****

Never/Rarely ****

**B3.** **Are you exercising 3 or more times per week, for at least 15 minutes each time, at the moment?**

Yes ****

No ****

*If NO, please go to question B4 below.*

**If YES, how long have you been exercising like this?**

Less than 30 days ****

1-6 months ****

7-12 months ****

Over a year ****

**B4.** **Are you seriously considering doing more exercise, and increasing your exercising to 3 or more times per week, for at least 15 minutes each time, during the next 6 months?**

Yes ****

No ****

*If NO, please go to Section C on the next page.*

If YES, how confident are you that you will start exercising at this rate during the next month?

Very confident ****

Somewhat confident ****

Not at all confident ****

Section C: Tobacco

*This section is about smoking cigarettes. Whether you are a smoker, ex-smoker or have never smoked, please answer the following questions.*

**C1.** **Which best describes you?** *(Please tick* ✓ *one box)*

I smoke every day ****

I smoke occasionally (less than once a day) ****

I used to smoke but I quit ****

I have never smoked ****

**C2.** **If you smoke cigarettes, how many do you usually smoke a day?** *(Please write a number)*

Number of cigarettes per day ________

**C3. If you used to be a smoker but have quit, how long ago is it since you stopped smoking?** *(Please tick* ✓ *one box)*

Less than 30 days ****

Less than 1 year ****

Over a year ****

Section D: Your Diet

*This section is about the foods that you normally eat. Tick the box after each food which best describes how you usually eat that food.*

***For example,*** *if you usually eat 2 slices of white bread per day you would fill in the box like this:*

| **Bread** | Never | 1 - 2  a day | 3 - 4  a day | 5 or more  a day |
| --- | --- | --- | --- | --- |
| White bread |  | ✓ |  |  |

***N.B. Note - if you never eat bread, please tick the ‘never’ box***

**D1.** **About how many pieces of bread do you eat on a usual day?**

**Are they usually white, brown, or wholemeal?**

*(Please tick  one box on* ***each*** line)

| **Bread** | Never | 1 - 2  a day | 3 - 4  a day | 5 or more  a day |
| --- | --- | --- | --- | --- |
| White bread |  |  |  |  |
| Brown sliced pan/bread |  |  |  |  |
| Wholemeal/soda bread or wholemeal scones |  |  |  |  |

**D2.** **About how many times a week do you have a bowl of breakfast cereal or porridge? What kind do you have most often?**

*(Please tick  one box on* ***each*** *line)*

| **Breakfast Cereal** | Never | 1 - 2  a week | 3 - 5  a week | 6 or more  a week |
| --- | --- | --- | --- | --- |
| **Sugar type**: e.g. Frosties or  **Rice/Corn type**: e.g.Corn Flakes |  |  |  |  |
| **Porridge** or Ready Brek or  **Wheat type**: e.g. Shredded Wheat or **Muesli type**: e.g. Alpen |  |  |  |  |
| **Bran type:** e.g. All-Bran |  |  |  |  |

**D3.** **About how many times a week do you eat a serving of the following foods?**

*(Please tick  one box on* ***each*** *line)*

|  | Never | 1 - 2  a week | 3 - 5  a week | 6 or more  a week |
| --- | --- | --- | --- | --- |
| Pasta or rice |  |  |  |  |
| Potatoes: baked, boiled, mashed |  |  |  |  |
| Peas |  |  |  |  |
| Beans (baked, tinned, dried)  or lentils |  |  |  |  |
| Other vegetables  (any type, fresh, frozen, tinned) |  |  |  |  |
| Fruit (fresh or canned) |  |  |  |  |

**D4.** **About how many times a week do you eat a serving of the following foods?**

*(Please tick ✓ one box on* ***each*** *line)*

|  | Never | 1 - 2  a week | 3 - 5  a week | 6 or more  a week |
| --- | --- | --- | --- | --- |
| Cheese (any except cottage) (include cheese dishes) |  |  |  |  |
| Beefburgers or sausages (include low fat) |  |  |  |  |
| Beef, pork or lamb (if vegetarian: nuts) |  |  |  |  |
| Bacon, meat pies, processed meat |  |  |  |  |

**D5.** **About how many times a week do you eat a serving of the following foods?**

*(Please tick ✓ one box on* ***each*** *line)*

|  | Never | 1 - 2  a week | 3 - 5  a week | 6 or more  a week |
| --- | --- | --- | --- | --- |
| Chicken or turkey |  |  |  |  |
| Fish (**NOT** fried)  (include tinned, baked, grilled) |  |  |  |  |
| **ANY** fried foods: fried fish, chips (include oven/micro),  cooked breakfast |  |  |  |  |
| Cakes, pies puddings, pastries,  ice cream |  |  |  |  |
| Biscuits (include butter and crackers), chocolate, crisps (include low fat varieties) |  |  |  |  |

**D6.** **About how much milk do you yourself use in a day, for drinking or in cereal, tea, or coffee? What kind of milk do you usually use?**

*(Please tick  one box on* ***each*** *line)*

|  | None | About a quarter pint | About a half pint | 1 pint or more |
| --- | --- | --- | --- | --- |
| Full cream |  |  |  |  |
| Semi-skimmed |  |  |  |  |
| Skimmed |  |  |  |  |

**D7.**  **About how many rounded teaspoons of margarine, butter or other spreads do you usually use in a day, for example on bread, sandwiches, toast, potatoes or vegetables?**

*(Please write the number of teaspoons in the boxes)*

**Butter or margarine**

(e.g. Flora, sunflower types,

teaspoons

Blue Band, Krona, Stork, Dairygold)

#### Low fat spread

(e.g. Low-Low, Outline, Golden Olive,

teaspoons

Shape, Flora Extra Light, Delight,

Benecol, Half Fat Butter, Dairygold light)

**D8.**  **What sort of fat do you use?**

*(Please tick ✓ one box on* ***each*** *line)*

| Fat Type | On bread and vegetables | For frying | For baking or cooking |
| --- | --- | --- | --- |
| Butter, dripping, lard, solid cooking fat |  |  |  |
| Hard or soft margarine |  |  |  |
| Polyunsaturated or sunflower  margarine or low fat spread |  |  |  |
| Pure vegetable oil (e.g. sunflower or olive) |  |  |  |
| I don’t use fat |  |  |  |

**D9.** **In an average day, how many portions (one fruit or an average sized serving) of fresh fruit or vegetables would you eat?** *(Please tick ✓ one box)*

None ****

1-3 a day ****

4 a day ****

5 or more a day ****

Section E: About You and Your Household

#### *This section contains some questions about you and your household.*

**E1.** **What age were you when you left school?** ________ years

**E2. What did your education include?** *(Please tick ✓ one answer)*

No schooling ****

Primary school education only ****

Some secondary education ****

Complete secondary education ****

Some third-level education at college, university, RTC ****

Complete third-level education at college, university, RTC ****

**E3. What is your current marital status?** *(Please tick ✓ one answer)*

Married/Living with partner **** Widowed ****

Separated/Divorced **** Single/Never married ****

**E4. What is your current employment situation?**

*(Please tick ✓ one answer)*

At paid work: Employee ****

At paid work: Self-employed ****

Homemaker ****

Unemployed, and looking for work ****

Student ****

Retired (having reached retirement age) ****

Unable to work due to sickness/disability ****

Other (please specify):

­­­­­­

**E5. What is your job title?**

(If you are not in a paid job at the moment, give title of your last job)

_________________________________________________

*If you are the principal wage earner in your household,*

*please go to Question E7 below.*

*If not, please answer Question E6*

*about the principal wage earner in your household:*

**E6. What is his/her job title?**

(If they are not in a paid job at the moment, give title of last job)

_________________________________________________

**E7. If you are a farmer, how many acres of land do you or your partner own?**

_________ acres

**E8. Do you have a medical card?**

Yes ****

No ****

# **E9.** We are interested in your journey to the doctor and to the hospital.

# Please complete the following table which asks about how you travel, how long it takes and how long you have to wait before being seen when you visit the doctor’s practice and the hospital.

| **Question** | The doctor’s practice | The hospital |
| --- | --- | --- |
| **How many miles** do you have to travel from your home to… |  |  |
| **How** do you usually travel to… (e.g. bus, car, taxi, walk) |  |  |
| **How long** does it usually take you to travel from your home to… | ______ minutes | _____ minutes |
| **How long** do you usually have to wait to see the doctor or nurse after you arrive at… | ______ minutes | _____ minutes |

# **E10.** We are interested to know how often you have attended your doctor or the hospital in the last 12 months, for any reason.

# Please write the number in the boxes below.

| Question | Your Answer |
| --- | --- |
| **How often** have you attended your GP in the **last 12 months**? |  |
| **How often** have you attended your practice nurse in the **last 12 months**? |  |
| **How often** have you attended hospital as an outpatient in the **last 12 months**? |  |
| **How often** have you been admitted to hospital as an inpatient in the **last 12 months**? |  |
| **How many days** have you spent in hospital in the **last 12 months**? |  |
| **How many times** have you visited the Accident and Emergency Department in the hospital in the **last 12 months**? |  |

Section F: Questions about How you Take your Medicines

*Many people find a way of using their medicines which suits them. This may differ from the instructions on the label or from what their doctor has said. We would like to ask you a few questions about how you use your medicines.*

Here are some ways in which people have said that they use their medicines.

For each of the statements, please tick ✓ the box which best applies to you.

| **Statement** | Always | Often | Sometimes | Rarely | Never |
| --- | --- | --- | --- | --- | --- |
| I forget to take my medicines |  |  |  |  |  |
| I alter the dose of my medicines |  |  |  |  |  |
| I stop taking my medicines for a while |  |  |  |  |  |
| I decide to miss out a dose |  |  |  |  |  |
| I take less than instructed |  |  |  |  |  |

Thank You!

You have now finished the questionnaire.

Thank you for taking the time to answer these questions.

When you are happy that you have answered all the questions,

please post the questionnaire back to your GP surgery

using the Stamped Addressed Envelope provided.

the SPHERE study is funded by

the Health Research Board


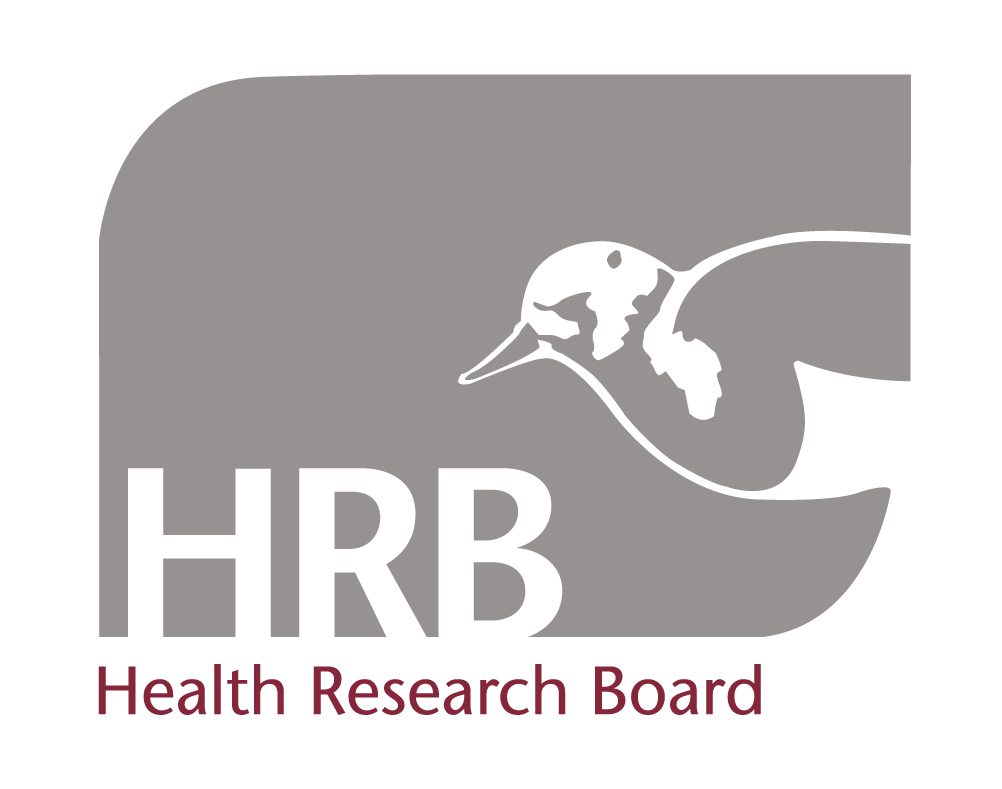

Supplement: Additional File 3 — Appendix C: Patient Questionnaire [file 1468-6708-6-11-S3.doc]
