# Supplementary material for: The SPHERE Study. Secondary prevention of heart disease in general practice: protocol of a randomised controlled trial of tailored practice and patient care plans with parallel qualitative, economic and policy analyses. [ISRCTN24081411]
Source: Curr Control Trials Cardiovasc Med. 2005 Jul 29;6(1):11. doi: 10.1186/1468-6708-6-11 (PMC1208929; doi:10.1186/1468-6708-6-11)
Supplement: Additional File 4 — Appendix D: Other Baseline Data Collection Documentation [file 1468-6708-6-11-S4.doc]

APPENDIX D: OTHER BASELINE DATA COLLECTION DOCUMENTATION

BASELINE Patient measurement record

BASELINE PATIENT HISTORY RECORD

BASELINE PATIENT MEASUREMENT RECORD

|  | **The SPHERE Study** **Secondary Prevention of Heart DiseasE in GeneRal PracticE** *A research study funded by the Health Research Board* |
| --- | --- |

**BASELINE Patient measurement record**

### *To be completed by the practice nurse or GP at patient’s first SPHERE consultation*

Patient Study ID: Patient Initials: Date of Birth:

Today’s Date: ___________________ Time at beginning of consultation: _______________

| Blood pressure | | / | 🞎 Right Arm  *(tick one)*  🞎 Left Arm |
| --- | --- | --- | --- |
| If B/P over 140/90 take a second reading at the end of the consultation | | / | 🞎 Right Arm  *(tick one)*  🞎 Left Arm |
| Total cholesterol — take today, if last cholesterol recorded more than 3 months ago | | mmol/l | |
| Fasting specimen taken?  🞎 Yes 🞎 No | If fasting specimen: HDL  LDL |  | |
|  | |
| Weight | | kg | |
| Height | | cm | |
| Waist measurement | | cm | |
| Hip measurement | | cm | |

| **Current Medications**  ***(covering the time period just BEFORE this consultation – NOT newly prescribed medication)*** | | | | | |
| --- | --- | --- | --- | --- | --- |
| Medication | Drug | Y/N | Specify | Dose | Contra Indication |
| Antiplatelet/  Anticoagulant | *Aspirin* |  |  |  |  |
|  | *Clopidrogel* |  |  |  |  |
|  | *Warfarin* |  |  |  |  |
| Lipid Lowering | *Statin* |  |  |  |  |
|  | *Fibrate* |  |  |  |  |
|  | *Esetimibe* |  |  |  |  |
| β Blocker | *β Blocker* |  |  |  |  |
| Antihypertensive | *Diuretic* |  |  |  |  |
|  | *ACE inhibitor* |  |  |  |  |
|  | *ARB* |  |  |  |  |
|  | *Calcium channel blocker* |  |  |  |  |
| Other Cardiovascular Drug |  |  |  |  |  |
|  |  |  |  |  |  |

Time at end of consultation: _______________

BASLEINE PATIENT HISTORY RECORD

|  | **The SPHERE Study** **Secondary Prevention of Heart DiseasE in GeneRal PracticE** *A research study funded by the Health Research Board* |
| --- | --- |

**BASELINE Patient HISTORY record**

Patient Study ID: Patient Initials: Date of Birth:

Today’s Date: ___________________

*(Leave shaded areas blank)*

| Sex | M F |  |  |
| --- | --- | --- | --- |
| GMS Card Holder | N Y N/A |  |  |
| Number of months since CHD diagnosis confirmed |  |  |  |
| Has patient had MI? | N Y | Tests confirming diagnosis: | ECG N Y  Enzymes N Y  Troponins N Y  Other N Y |
| Months since most recent MI |  |  |  |
| If more than one MI recorded – how many? |  |  |  |
| Angina diagnosed | N Y | Tests confirming diagnosis: | ECG N Y * n/r  Scan N Y n/r  EST N Y n/r  Angiogram N Y n/r |
| Record of CABG | N Y | Total Number |  |
|  |  | Date of last |  |
| Record of PTCA | N Y | Total Number |  |
|  |  | Date of last |  |
| Record of Diabetes | N Y | Type | Type I N Y  Type II N Y  Pre-diabetes N Y |
|  |  | Confirmed by FBG/GTT | N Y |
| Number of visits to GP in previous 6 months  (face to face consultation only, not repeat prescribing) | Visits: |  |  |
| Number of visits to practice nurse in previous 6 months  (for any reason) | Visits: |  |  |
| Number of visits to outpatient clinics in last 12 months (for any reason) | Visits: |  |  |

* n/r – No record found

| Number of times admitted to hospital in last 12 months (for any reason) | Admissions: |  |  | |
| --- | --- | --- | --- | --- |
| Number of days spent as inpatient in last 12 months  (for any reason) | Days: |  |  | |
| Number of visits to A&E Dept (for any reason) | Visits |  |  | |
|  |  |  |  | |
| **Risk Factors Recorded:** |  |  |  | |
| CIGARETTE SMOKING STATUS RECORDED?  **In the last 12 months**  **Recorded ever** | N Y  N Y | DATE of last record: | Last recorded status:  🞎 Never smoked  🞎 Ex-smoker  🞎 Smoker  If smoker – how many cigarettes per day? | |
| CHOLESTEROL LEVEL RECORDED?  **In the last 12 months** | N Y | DATE of last report: | VALUES: | |
| BLOOD PRESSURE RECORDED?  **In the last 12 months** | N Y | DATE of last record: | VALUE: | |
| BLOOD GLUCOSE RECORDED?  **Recorded ever** | N Y | DATE of last record: | Fasting  Random | Result: |

* n/r – No record found

Recorded by ___________________________

Research Nurse
